# Developing Global Competence From Participation in an Online Collaborative Multicultural Learning Experience

**DOI:** 10.1177/01939459261488136

**Published:** 2026-10-13

**Authors:** Gulzar Malik, Arkers Kwan Ching Wong, Jacqueline Parkin, Jacqueline Johnston, Kusrini Kadar, Katherine Frodsham Webster

**Affiliations:** 1 School of Nursing & Midwifery, La Trobe University, Melbourne, VIC, Australia; 2 School of Nursing, The Hong Kong Polytechnic University, Hong Kong; 3 Department of Nursing and Healthcare, Leeds Beckett University, UK; 4 Universitas Hasanuddin, Indonesia, Ajman University, UAE; 5 Department of Care Science, Malmö University, Sweden

**Keywords:** COIL, internalisation at home, global competence, global health, nursing and midwifery education, nursing students

## Abstract

**Objectives:**

This innovative Collaborative Online International Learning (COIL) program aimed to enhance undergraduate nursing and midwifery students’ global health knowledge, cultural awareness, and global competence by connecting students and educators from multiple countries.

**Methods:**

Five international universities co-designed the program, delivering 5 online webinars incorporating global health themes and cultural activities. Retrospectively linked REDCap pre- and post-program surveys enabled within- participant analysis. As questionnaire data were ordinal and non-normal, medians and interquartile ranges (IQRs) were reported, with Wilcoxon signed-rank and exact McNemar tests used. Student evaluations explored learning experiences and perspectives.

**Results:**

Thirty-one of 45 enrolled students (69%) completed both questionnaires. Significant improvements were observed in Perspective Taking and Adaptability (median: 28 [IQR 25-29] vs 29.5 [IQR 28-35]; median change: 3 [IQR 0-6]; *P* = .002) and Global-Mindedness (16 [IQR 15-18] vs 19 [IQR 15-20]; median change: 0.5 [IQR 0-3]; *P* = .008). Awareness of intercultural communication increased but was not statistically significant at the subscale level. No baseline differences were identified by gender, university/region, year level, or completion status. Student evaluations indicated greater confidence engaging with culturally diverse individuals and enhanced inclusivity, adaptability, and openness to different perspectives.

**Conclusion:**

Participation in this COIL program was associated with improvements in key dimensions of global competence, particularly perspective taking, adaptability, and global-mindedness. Students valued the cultural learning and international peer engagement, supporting COIL as a practical internationalisation-at-home strategy. Future research should address questionnaire attrition and examine the sustainability of these gains over time.

## Introduction

Globalisation has created a need for healthcare professionals to collaborate and work in a multicultural environment. This requires professionals to be interculturally competent in providing high-quality care to patients and communities they serve. However, the knowledge and skills that are essential to be culturally competent are difficult to teach and learn in the traditional classroom. Studies suggest the most effective way these attributes and skills are acquired is through global learning.^1,2^

Many global universities have devoted resources towards offering internationalisation experiences to nursing and midwifery students. Study abroad programs offer opportunities to develop cultural awareness and global competence among nursing students.^3^ When Covid-19 disrupted international travel worldwide, Collaborative Online International Learning (COIL) emerged as the most cost-effective approach and gained attention to achieve outcomes in global learning.^4^

The Organisation for Economic Cooperation and Development (OECD) Programme for International Student Assessment^5^ defines global competence as “the capacity to examine local, global and intercultural issues, to understand and appreciate the perspectives and world views of others, to engage in open, appropriate and effective interactions with people from different cultures and to act for collective well-being and sustainable development.”^6^ Acquiring global competence is a lifelong learning process; to achieve this, educational institutions play an important role in educating and helping students to develop global competence by embedding international learning in their curriculum using in-country experience and/or COIL.^7^

In nursing and midwifery education, COIL pedagogy has become popular in the last few years. Nurse academics who have developed innovative programs using the COIL approach reported that this pedagogy enables meaningful multi-institution exchanges between academics and students.^4^ It provides students with an opportunity to interact, connect and take ownership of their learning in a multicultural learning environment.^8^ Interactions with colleagues residing in other countries promote cross-cultural awareness,^9^ communication skills,^2^ teamwork, critical thinking^10^ and collaboration skills.^11^

To date, COIL program outcomes have been published in the context of other healthcare disciplines, however, the design of COIL programs and the associated outcomes in the context of nursing and midwifery education are limited. This research aimed to fill the gap by reporting a unique COIL program involving multiple partner universities and its outcomes in acquiring global competence among undergraduate nursing and midwifery students.

## Research Questions

The primary research question guiding this study was: Does participation in a five-week, multicultural COIL program improve undergraduate nursing and midwifery students’ global competence, and what were students’ experiences of the program? This was operationalised through 2 specific questions:

1. What are nursing and midwifery students’ experiences of attending a multicultural COIL program?
2. What are nursing and midwifery students’ knowledge, skills and attitudes in developing global competence pre- and post-program?

## COIL Program

This COIL program *Learning Global Health: An Online Collaborative Multicultural Learning Experience* aimed to enhance undergraduate nursing students’ knowledge of global health, develop cultural awareness, and offer opportunities to gain knowledge, skills and attitudes to acquire global competence by bringing international partners and students from around the world.

### Partnership Formation and Co-Design

The program was conceptualised by the lead investigator and content developer at the host Australian university, who had been awarded internal university funding for an internationalisation-at-home initiative. An Expression of Interest was sent to existing institutional partners selected for their cultural and contextual diversity. Universities in Indonesia, Hong Kong, Sweden and the United Kingdom accepted the invitation. The 5 participating universities were chosen because (a) they each include intercultural competence and global health as graduate-level outcomes within their nursing/midwifery curricula, and (b) they represent 4 distinct cultural and healthcare contexts. The program was co-designed through a series of monthly online planning meetings held over 6 months prior to delivery. Curriculum content and learning objectives were negotiated collectively among academic leads from the 5 partner universities, with each partner contributing the focal theme that best reflected its national context and academic strengths. The themes of the webinars included community health in the context of Indonesia, family-centred care, collaborative leadership in a healthcare setting, and advanced nursing practice. The 4 core themes that emerged from the co-design process were mapped to the 4 dimensions of the OECD/PISA Global Competence Framework (see Table 1).

**Table 1.** Mapping of Co-Design Themes to OECD/PISA Global Competence Dimensions and Program Outcomes.

Webinar theme	Hosting partner	OECD/PISA dimension	Program outcomes
Community health (Indonesia)	Indonesia	Examine issues of local, global and cultural significance- comparing health practices across 5 countries	Acquire knowledge of global health
Family-centred care	UK		
Collaborative leadership	Sweden	Understand and appreciate diverse perspectives and worldviews	Develop cultural awareness
Advanced nursing practice	Hong Kong	Engage in open, appropriate and effective intercultural interactions	Gain knowledge, skills and attitudes to acquire global competence
		Take constructive action toward sustainable development and collective well-being	

Abbreviations: OECD, Organisation for Economic Cooperation and Development; PISA, Program for International Student Assessment.

### Webinar Structure

The program comprised 5 online webinars, each of two-hour duration, conducted weekly over 5 consecutive weeks. The host Australian university led overall coordination, while each of the first 4 webinars was hosted and chaired by a different partner university. Using the concept of a human library, experts from the fields were invited from the host country to discuss a health topic and share research/expertise on the chosen theme.

To accommodate participating universities across multiple time zones, all webinars were scheduled at a single time window agreed by all partners (early evening UK time/late evening AEST), which was workable for all partner countries. English was used as the lingua franca for all program activities; this was confirmed as the language of instruction at each partner university, and therefore translation tools were not required.

The activities in each webinar were structured as follows:

- Theme focused keynote presentation: 40 minutes
- Human library/Meeting with experts: 30 minutes
- Language Class/Dance Class/other cultural activity: 30 minutes
- Group interaction time via breakout rooms: 15 minutes.

#### Group Composition

After registrations closed, the program coordinator allocated students into 4 pre-arranged multicultural breakout groups, ensuring that each group contained students from each of the 5 partner universities (where numbers permitted). Group membership remained stable across the 5 webinars to support relationship-building, peer trust and continuity of discussion across the program.

#### Case Studies and Guided Questions

Each multicultural group was assigned 1 case scenario aligned to the 4 core themes of the program. The case studies were developed by the program leads from each partner university and were paired with 5 to 6 guided questions. The guided questions were designed using the 4 dimensions of the OECD/PISA Global Competence Framework (examining issues of local/global/cultural significance; understanding diverse perspectives; engaging in respectful intercultural interactions and acting for collective well-being).

Students received the case scenarios immediately following the fourth webinar and were given 2 weeks to prepare their group analysis prior to the fifth webinar. Groups self-organised their preparatory discussions through online platforms (Zoom, WhatsApp, email). Immediately before the final presentations, students were placed in their breakout groups for a further 20-minute preparatory session to ensure equitable readiness regardless of pre-meeting attendance. In the fifth webinar, each group presented its case analysis and a group reflection on the program. An example of one case study with its full set of guided questions is provided as a Supplemental File.

## Methods

### Design

The study employed a pre-post survey design that explored participating students’ knowledge, skills and attitudes in acquiring global competence. Both quantitative (Global Competence Questionnaire) and qualitative (open-ended component of evaluation responses) data were collected and integrated to provide a comprehensive view of program outcomes. In addition, after each webinar, students also completed the evaluation that contained both closed-ended and open-ended questions. The reporting of this study conforms to the TREND (Improving the Reporting Quality of Non-Randomised Evaluations of Behavioural and Public Health Interventions) statement.^12^

### Recruitment

Using convenience sampling, all nursing and midwifery students enrolled in the Bachelor of Nursing and/or Bachelor of Midwifery programs at the participating universities were invited to participate in the COIL program and its associated research component, conducted virtually (online) in July 2023.

At one of the participating Australian universities, a double-degree program offers a dual qualification in nursing and midwifery, with the first 2 years comprising shared nursing subjects; students enrolled in either the Bachelor of Nursing or the combined Bachelor of Nursing/Bachelor of Midwifery were therefore both eligible. The COIL program addressed global health and cultural competence themes that are equally relevant to both professions, supporting their inclusion together.

A program flyer and a link to the program video with all relevant information were shared with students. Each partner university posted the information on its university intranet page and/or its Learning Management System. A registration link to the program was embedded in the poster for students to register their intention to attend the program. Regarding the research, the Chief Investigator sent the information as well as the Participant Information Sheet (PICF) to all registered participants via email before the program.

### Data Collection

Data were collected via 2 sets of anonymous questionnaires. At the beginning of the first webinar and at the conclusion of the last webinar, students were asked to complete a Global Competence Questionnaire that measured their knowledge, skills, and attitudes in acquiring global competence. Demographic information was also collected regarding students’ age, gender, university and study year. Pre-and post-questionnaires were administered through an anonymous REDCap link delivered via the Zoom chat function and email; responses were required approximately 10 to 15 minutes to complete. Pre/post responses were matched using a self-generated participant code. Data were stored on the host university’s password-protected research drive accessible only to the research team.

After each webinar, students were asked to complete the evaluation form to capture their experiences. This questionnaire included both closed- and open-ended questions. A link to the evaluation form was shared using the Zoom chat function and via email, in case students had to leave early during the webinar. This questionnaire contained 5 closed Likert-scale items and 3 open-ended questions. The post-program evaluation distributed at the end of the fifth webinar additionally included reflective open-ended items.

### Ethical Considerations

Ethics approval (HEC22073) was obtained from the Australian tertiary institution to conduct the study. Respective partner universities received permission from their universities. Participation in the study was voluntary and students who attended the program had a choice to participate or not to participate in the research. Informed consent was implied through the voluntary completion of the Global Competence questionnaire and program evaluation.

### Global Competence Questionnaire

Underpinned by a Global Competence Framework, The Program for International Student Assessment (PISA)^5^ designed a Global Competence Questionnaire. This tool was developed for academic institutions aiming to assess global competence in students from educational programs. Effective education for global competence allows students to mobilise and use their knowledge, skills, attitudes and values during an exchange of ideas on global issues while interacting with people from different cultural backgrounds.^6^

As per the PISA global competence framework, global competence has 4 dimensions: (1) the capacity to examine issues of local, global, and cultural significance; (2) the capacity to understand and appreciate different perspectives and worldviews; (3) the ability to establish positive interactions with people and (4) the capacity to take constructive action toward sustainable development and collective well-being. These dimensions are strongly interdependent and are supported by 4 key factors comprised of knowledge, skills, attitudes and values. For example, examining a global issue requires knowledge of a particular issue, the skills to transform this awareness into a deeper understanding, and attitudes and values to reflect on the issue from multiple cultural perspectives.^5^ The purpose of assessing global competence using the above-mentioned 4 factors is to elicit how well the students are prepared to examine contemporary issues of local, global and intercultural significance as well as an ability to acknowledge and accept various perspectives.^5^

The Global Competence Questionnaire comprised 14 subscales. In our study, 6 subscales (33 items in total) were selected because they map directly onto the 4 dimensions of the OECD/PISA framework and to the program objectives, namely: (1) Contact with people from other countries (4 statements; dichotomous); (2) Interest in learning about other cultures (5 statements, Cronbach’s α = 0.82); (3) Respect for people from other cultural backgrounds (5 statements, Cronbach’s α = 0.85); (4) Awareness of intercultural communication (7 statements, Cronbach’s α = 0.88); (5) Perspective-taking and Adaptability (7 statements, Cronbach’s α = 0.89) and (6) Global-mindedness (5 statements, Cronbach’s α = 0.79). The remaining subscales (eg agency, attitudes towards immigrants, language proficiency) were beyond the scope of a 5-week webinar series and were therefore omitted.

The subscale mean scores and the total score were calculated in the latter 5 subscales while no scores were computed in the “Contact with people from other countries” subscale since all statements triggered dichotomous answers. The subscale mean and total scores were the average scores and sum of scores obtained from each statement in the respective subscale. For the subscales of “Interest in learning about other cultures,” “Respect for people from other cultural backgrounds” and “Perspective taking and adaptability” a score was given to an answer of “not at all like me” (1 point), “not much like me” (2 points), “somewhat like me” (3 points), “mostly like me” (4 points) and “very much like me” (5 points), with higher scores representing a higher level of global competence in the respective domain. The highest score for “Interest in learning about other cultures” and “Respect for people from other cultural backgrounds” subscales were 25, whereas the highest score for “Perspective taking and adaptability” was 35.

Similarly for another 2 subscales, “Awareness of intercultural communication” and “Global-mindedness,” 1 point was awarded for a response of “strongly disagree” towards a statement, 2 points for “disagree,” while 3 points were given for an answer of “agree” and 4 points for “strongly agree.” Participants who got higher scores were more likely to possess a higher awareness of intercultural communication and a stronger sense of global-mindedness. The highest scores for the 2 named subscales were 28 and 20, respectively.

### Data Analysis

Data were analysed using SPSS version 26 (IBM Corp., Armonk, NY, USA). The pre-program and post-program survey files did not retain a shared participant identifier. Accordingly, matched pre-post cases were reconstructed retrospectively using stable participant characteristics unlikely to change during the program, including age category, gender, university, year level, degree, previous short study tour or international exchange experience, previous COIL experience and the 4 dichotomous items on contact with people from other countries. This process yielded 31 reconstructed matched cases for within-participant analyses.

Descriptive statistics were used to summarise demographic characteristics. Because the questionnaire data were ordinal and non-normal, pre-program and post-program total scores for the 5 scored subscales were summarised using medians and IQRs and compared using Wilcoxon signed-rank tests. Exact McNemar tests were used for the 4 dichotomous contact items. In addition, exploratory subgroup analyses of pre-test subscale totals were conducted using Mann-Whitney *U* tests for gender and Kruskal-Wallis tests for university/region and year level. To examine attrition bias, matched completers were compared with pre-test-only respondents using chi-square tests for categorical variables and Mann-Whitney *U* tests for baseline subscale totals. Statistical significance was set at *P* < .05.

Post-program evaluations were analysed using frequency distributions, and open-ended evaluation responses were analysed using Braun and Clarke’s^13^ six-phase reflexive thematic analysis framework: (1) data familiarisation through repeated reading; (2) generation of initial codes through line-by-line inductive coding; (3) collation of codes into candidate themes; (4) review of themes against the dataset; (5) definition and naming of final themes and (6) production of the report with illustrative quotations. Two researchers independently coded the dataset, met to reconcile codes through iterative discussion, and developed candidate themes that were subsequently reviewed and finalised by the full research team. A reflexivity log was maintained throughout the analysis to support trustworthiness.

## Results

### Characteristics of Participants

A total of 31 of 45 enrolled students (69%) completed the questionnaires. Using the survey exports, all 31 post-program records were linked to corresponding pre-program records and formed the analytic sample for within-participant analyses. Within this matched sample, 28 participants (90.3%) were female, 2 (6.5%) were male and 1 (3.2%) had missing gender data. Most participants were aged between 18 and 30 years (n = 25, 80.6%). Participants represented all 5 collaborating universities, with the largest proportion from The Hong Kong Polytechnic University (n = 9, 29.0%), followed by Hasanuddin University (n = 7, 22.6%), La Trobe University (n = 6, 19.4%), Sheffield Hallam University (n = 6, 19.4%) and Malmo University (n = 3, 9.7%). Students were enrolled across all 4 year levels. Most reported no previous short study tour or international exchange experience (25/31, 80.6%; 1 missing) and no prior COIL or virtual mobility experience (27/31, 87.1%). Table 2 summarises the participant characteristics of the analytic sample.

**Table 2.** Participant Characteristics of the Matched Analytic Sample (n = 31).

Characteristic	Category	n	%
Gender	Male	2	6.5
	Female	28	90.3
	Missing	1	3.2
Age (years)	18-20	10	32.3
	21-30	15	48.4
	31-40	3	9.7
	41-50	2	6.5
	51-60	1	3.2
University/region	Australia (La Trobe University)	6	19.4
	Indonesia (Hasanuddin University)	7	22.6
	Hong Kong (The Hong Kong Polytechnic University)	9	29.0
	Sweden (Malmo University)	3	9.7
	United Kingdom (Sheffield Hallam University)	6	19.4
Year level	Year 1	7	22.6
	Year 2	7	22.6
	Year 3	10	32.3
	Year 4	7	22.6
Previous short study tour/international exchange	Yes	5	16.1
	No	25	80.6
	Missing	1	3.2
Previous COIL / virtual mobility experience	Yes	4	12.9
	No	27	87.1

### Attrition and Baseline Comparisons

Questionnaire attrition from the pre-program survey to the post-program survey was 39.2% (20/51). Relative to total program attendees, 31 of 45 students (68.9%) contributed post-program quantitative data. Exploratory baseline analyses within the matched analytic sample detected no statistically significant differences in any pre-test subscale total by gender, university/region or year level. Because the primary quantitative analyses were within-person nonparametric comparisons and no baseline subgroup imbalance was identified, no additional covariate adjustment was undertaken.

Comparisons between matched completers and pre-test-only respondents also showed no statistically significant baseline differences in age category (χ^2^ = 1.98, *P* = .74), gender (χ^2^ = 3.55, *P* = .31), university/region (χ^2^ = 6.68, *P* = .15), year level (χ^2^ = 3.40, *P* = .33), previous short study tour or international exchange experience (χ^2^ = 1.05, *P* = .30), or prior COIL or virtual mobility experience (χ^2^ = 0.00, *P* > .99). Baseline subscale totals likewise did not differ significantly between completers and non-completers: Interest in Learning About Other Cultures (median, 23 vs 22.5; *U* = 345.5; *P* = .49), Respect for People From Other Cultural Backgrounds (25 vs 25; *U* = 306.0; *P* = .91), Awareness of Intercultural Communication (23 vs 23.5; *U* = 238.5; *P* = .63), Perspective Taking and Adaptability (28 vs 30.5; *U* = 234.5; *P* = .15), and Global-Mindedness (16 vs 17; *U* = 266.5; *P* = .51). These findings reduce, but do not eliminate, concern regarding attrition bias.

### Pre-Test, Post-Test and Change Scores on the Modified Global Competence Questionnaire

Table 3 presents the pre-program, post-program, and change scores for the 5 scored subscales using medians and IQRs. Statistically significant improvements were observed for Perspective Taking and Adaptability (pre-test: 28 [IQR: 25-29] vs post-test: 29.5 [IQR: 28-35]; median change = 3 [IQR: 0-6]; *W* = 45; *z* = −3.17; *P* = .002; *r* = 0.63) and Global-Mindedness (16 [IQR: 15-18] vs 19 [IQR: 15-20]; median change = 0.5 [IQR: 0-3]; *W* = 25; *z* = −2.65; *P* = .008; *r* = 0.62). Changes in Interest in Learning About Other Cultures, Respect for People From Other Cultural Backgrounds, and Awareness of Intercultural Communication were not statistically significant, although awareness scores showed a descriptive upward trend. For the 4 dichotomous contact items, exact McNemar tests showed no statistically significant pre-post changes in contact with people from other countries in the family (*P* > .99), at university (*P* = .63), in the neighbourhood (*P* = .50) or in the circle of friends (*P* = .69).

**Table 3.** Pre-Program, Post-Program, and Change Scores for the 5 Scored Subscales.

Subscale	n	Pre-test median (IQR)	Post-test median (IQR)	Change median (IQR)	*W*	*z*	*P*	*r*
Interest in learning about other cultures	31	23 (20.5-25)	24 (20-25)	0 (−0.5-1)	97	−0.30	.76	0.07
Respect for people from other cultural backgrounds	30	25 (22.5-25)	25 (21.5-25)	0 (−1.5-1)	75	−0.46	.64	0.11
Awareness of intercultural communication	29	23 (21-26)	25 (21.5-28)	1 (−1-4)	112.5	−1.61	.11	0.32
Perspective taking and adaptability	30	28 (25-29)	29.5 (28-35)	3 (0-6)	45	−3.17	.002	0.63
Global-mindedness	30	16 (15-18)	19 (15-20)	0.5 (0-3)	25	−2.65	.008	0.62

Abbreviation: IQR, interquartile range.

### Illustrative Item-Level Shifts

Although the revised inferential analyses prioritise subscale-level outcomes, the item distributions showed patterns consistent with improved intercultural confidence. For example, the number of participants selecting “strongly agree” for “I frequently check that we are understanding each other correctly” increased from 12 at pre-test to 19 at post-test. For “I give concrete examples to explain my ideas,” “strongly agree” responses increased from 8 to 16, and for “I explain things very carefully,” they increased from 9 to 16.

The clearest descriptive shifts were observed in the Perspective Taking and Adaptability domain. For adapting to different situations under stress, the number of participants endorsing “very much like me” increased from 4 to 15. For adapting easily to a new culture, the corresponding count increased from 6 to 15, and for overcoming difficulties in interacting with people from other cultures, it increased from 6 to 14. In the Global-mindedness domain, “strongly agree” responses increased from 8 to 19 for feeling responsible to act when seeing poor health conditions around the world, and from 11 to 17 for perceiving that one’s behaviour can affect people in other countries. These item-level shifts are presented only to illustrate the direction of change and should not be interpreted as stand-alone confirmatory findings.

### Post-Program Evaluations

A total of 45 students participated in the COIL program, of whom 31 completed the program evaluation. Overall, students rated their experience across the 5 webinars highly, with a mean score of 8.9/10 on a Likert scale (0 = lowest, 10 = highest). They also reported positive experiences connecting with peers from partner universities, with a mean score of 8.3/10. All participants agreed or strongly agreed that the COIL experience exposed them to new perspectives on culture and diversity. Furthermore, 98% agreed or strongly agreed that the program enhanced their knowledge and skills relevant to nursing practice and future careers. A key highlight identified by respondents was the opportunity to collaborate and work with students from partner universities, which 96% described as invaluable.

When participants were asked to reflect on how participation in the webinars influenced their current and future nursing practice, and to share their overall experience of the program, students’ responses converged into 4 key themes: cultural knowledge and competence; skills and behaviours; practice horizons and insights for improving future COIL initiatives.

#### Cultural Knowledge and Competence

A large number of students appreciated the cultural perspectives they gained from the webinars delivered by the participating universities on various topics. As one student reflected, “*a new perspective on healthcare from another country and some cultural perspectives which are different to my country and culture I live in*” (student 5). Some students highlighted that in today’s globalised world, the opportunity to connect with peers and faculty members from different countries fosters cultural awareness, but most importantly, cultural competence which is an essential aspect of nursing practice at present and in future as evidenced by “*it has opened my eyes to different cultural aspects and becoming aware of cultural sensitivities will enhance my future nursing practice*” (student 11). Another student echoed “*gaining cultural competence is important while working as a community nurse. Learning intercultural communication is necessary*” (student 18).

Participating students recommended continuing COIL experiences, as this helped them develop greater cultural competence. One student shared “*COIL helped me to learn about being culturally competent that requires empathy, understanding, kindness and offering care that is aligned to your client’s cultural values*” (student 21). Another student reflected: “*my patients might come from a different culture than myself and therefore might have other expectations of the interaction*” (Student 14). A further student commented that the webinars “*remind me to always respect people from various cultural backgrounds and being sensitive and respectful to their cultures*” (Student 10).

#### Skills and Behaviours

Most students reflected that participation in the webinars had a positive impact on their skills and behaviours. Their responses indicated improvements in communication, confidence, leadership, inclusivity, adaptability, cultural competence.

Several comments related specifically to communication, for example, one student stated, “*l am open minded that it might not work for everyone however if the communication with the patient is good from the beginning you will be able to tell if that might be the right approach*” (student 19). Another student reflected that the webinars had “*positively impacted effective communication*” (student 09). This aligns with the results of global competence questionnaire, in which students demonstrated improvement and placed value on intercultural communication. Evidence of increased inclusivity was also reflected in students’ comments, such as “*I’ll be more conscious about dad/partner’s well-being in pre/postnatal care and be sure to include them in the care plan*” (student 6) and “*Reflect on inclusive language*” (student 8). Participants’ excerpts also demonstrated growth in perspective-taking and adaptability, with students showing measurable improvement in these domains following the program.

#### Practice Horizons

The webinars provided students with an opportunity to reflect on the role of a nurse and healthcare approaches across the participating countries. Comments included “*I understand the differences of nursing practice from different countries*” (student 25). Another student shared “*I am beginning to see nursing from a broader perspective*” (student 14). One student described how participating in the webinars influenced her future career aspirations: “*piquing my interest to work overseas*” (student 21). Additionally, a student reflected on learning from a perinatal webinar, noting that “*a better awareness of men’s mental health is needed to better support men during/post pregnancy*” (student 30).

#### Insights to Improve Future COIL

In the program evaluation, participants provided invaluable insights into ways the future COIL program can be enhanced. Most participants highlighted that the connection and engagement time with other students in each of the webinars was insufficient. Based on the themes of each webinar, students were given a few questions to discuss in breakout rooms with multicultural peers during the final 15 minutes. Several participants suggested, “*longer time should be dedicated to interacting with students. Spending 10-15 minutes in each webinar wasn’t sufficient*” (student 7). Another student raised “*some students took time to open up in the breakout rooms, more time was needed to discuss the questions and to know each other*” (student 10). Some participants proposed reducing the duration of keynote speakers or human library research sessions to allow for more interaction time, as evidenced by “*time for group discussion need to extend and organisers should look into the time allocated for other presentations*” (student 14). Similarly, another participant shared “*I prefer to have a longer group discussion rather than listening to presenters as we learnt more connecting with other students*” (student 16).

Another recommendation that emerged from the program evaluation was time management. Some participants, particularly those who were attending the webinars in the evenings, raised concerns about their duration. As the webinars incorporated keynote speakers, human library research sessions, cultural activities and student interactions, 2 webinars exceeded the dedicated 2 hours. One student suggested that “*It would have been appreciated if webinars stayed within 2 hours, it was difficult to concentrate for more than 2 hours without break*” (student 12).

## Discussion

Study abroad programmes have been shown to enhance cultural awareness in nursing students,^14^ however overseas experiences for nursing and midwifery students can be challenging to arrange which require considerations of cost of travel, personal and family circumstances, language proficiency requirements, and course demands and regulations.^8,15^ In the last few years, COIL programs have provided an alternative approach to enhance students’ intercultural competence using a virtual platform.^3,16^ Our COIL program brought 5 universities’ faculty members and students together intending to develop nursing and midwifery students’ global competence. Most of the students involved in this program had no prior experience of taking part in an international exchange or COIL activity. In the nonparametric analyses, the significant improvements were observed in perspective taking and adaptability and in global-mindedness, whereas awareness of intercultural communication showed a favourable descriptive trend but did not achieve statistical significance at the subscale level. These findings suggest that the program’s pedagogical design emphasised reflective dialogue and adaptability rather than expanding social contact networks, which is consistent with no change in the dichotomous Contact subscale; and the relatively short 5-week program may be insufficient to shift broad attitudinal constructs (Respect, Interest) but is sufficient to shift situation-specific intercultural skills and global-mindedness. This shows short-term virtual exchange may be particularly effective in broadening students’ willingness to engage with different perspectives and in strengthening a sense of global professional responsibility.

This study also suggested that most students valued the opportunity to make connections and work collaboratively with other students from partner universities. Students were provided with a range of different topics relevant to their clinical practice. The topics selected challenged their thinking and perspectives of global issues relevant to healthcare, and provided them with the opportunity to explore the knowledge and skills that would be useful for their nursing practice and future careers. Reflecting on their practice can be transformational, leading to an increased self-awareness and a greater understanding of various cultures that exist globally.^17^ The benefits of connecting with other students from different cultures were supported by Limoges et al^18^ who explored how globally-networked learning influenced nursing students’ understanding of health and nursing in Canada and Denmark. They found that connecting to other students enhanced their understanding of the influence of culture on nursing practice. This kind of interaction can lead to a deeper reflection of one’s practice and aid the development of culturally safe care.^19^

Despite these positive outcomes, questionnaire attrition was substantial. Although completer–dropout comparisons did not identify statistically significant baseline differences in demographic characteristics or pre-test subscale totals, the possibility of attrition bias due to unmeasured differences in engagement, motivation, or scheduling constraints remains. Participants also expressed dissatisfaction with the limited time available in breakout rooms to engage with peers. They reflected that additional time for peer interaction and relationship building would have enhanced the learning experience. Future COIL events would benefit from a shift in focus from tutor-led activities to more student-led approaches, for example working through case studies. This is supported by Stärke^20^ who suggested that students value time collaborating with other students therefore, tutors who deliver virtual exchange programmes should recognise the essential role in facilitating this as well as delivering academic content.

Overall, the program provided students with valuable opportunities to deepen their understanding of culture, nursing, and healthcare within both their own and partner countries. It fostered reflection on the knowledge, skills and attributes required to function as healthcare practitioners and global citizens, while encouraging students to adopt more culturally sensitive approaches to their professional practice.

### Recommendations

Recommendations arising from this program emphasise that the successful implementation of COIL initiatives requires careful consideration and strategic planning. The selection of international partners and student cohorts should be aligned with the program’s objectives to ensure meaningful collaboration and outcomes. One of the key challenges identified was managing global time zone differences, which can hinder effective communication and engagement. Addressing this issue through proactive planning, flexible scheduling, and close coordination with partner universities is essential to the program’s ongoing success.

The continuity of COIL programs emerges as a critical factor, emphasising the necessity of providing equal opportunities for nursing and midwifery students to engage with the international aspects of the curriculum over an extended period. Additionally, effective time management is highlighted, emphasising the importance of keeping the duration of webinars within the stipulated time frame to optimise engagement and accommodate diverse schedules.

Notably, the incorporation of cultural activities, which gained high appreciation from students, should be sustained in future programs, enriching the intercultural learning experience. Clear communication of expectations regarding program requirements and assessment tasks is deemed essential for student understanding and preparedness. Finally, recognising the significance of fostering connectedness among students, adequate time should be allocated for group discussions, promoting a sense of community and collaboration within the virtual learning environment.

Moving forward, it is imperative to consider the integration of multi-disciplinary programs alongside nursing and midwifery-focused initiatives. Recognising the interconnected nature of healthcare professions, future COIL programs should be designed to foster collaboration among students from diverse disciplines, promoting a holistic understanding of global health challenges and solutions.

Students’ evaluations suggested that future COIL programs should prioritise opportunities for active peer-to-peer engagement, support critical reflection on practice, and foster the development of culturally safe care. Ongoing evaluation of virtual exchange initiatives such as COIL is essential, with participant feedback used to guide iterative improvements. Future longitudinal studies are needed to assess the sustained impact of COIL participation on students’ global competence. Program structures should be refined to align with student preferences and evolving needs, ensuring continuous enhancement and adaptability to diverse educational contexts. As technology and pedagogy continue to advance, COIL programs must remain dynamic and responsive, integrating innovative teaching methodologies and emerging technologies to further enrich the virtual learning experience.

### Limitations

The findings of this study should be interpreted in light of several limitations. First, the study used a single-group pre-post design with voluntary participation, which limits causal inference and may have introduced self-selection bias. Second, questionnaire attrition was substantial: only 31 of 51 baseline respondents completed the post-test survey. Although completer–dropout comparisons did not identify statistically significant baseline differences in measured demographic characteristics or baseline subscale totals, unmeasured differences in motivation, engagement, scheduling constraints or digital access may still have influenced follow-up participation. Third, the archived pre-program and post-program survey exports did not retain a direct participant identifier, so matched cases had to be reconstructed retrospectively from stable participant characteristics. Although this linkage process was conservative and one-to-one, some degree of matching error cannot be ruled out. Fourth, the modified questionnaire relied on self-report and included only 6 of the 14 subscales in the original OECD instrument. We therefore analysed item-level changes descriptively only and based the primary inference on multi-item subscale totals. Finally, the study evaluated short-term outcomes only; longer-term follow-up would be needed to determine whether the observed improvements are sustained and whether they translate into practice behaviour.

## Conclusion

This study suggests that COIL can support the development of selected dimensions of global competence among nursing and midwifery students, particularly perspective taking, adaptability and global-mindedness. Participants also described meaningful cultural learning and valued engagement with peers from partner universities, indicating that virtual international collaboration can enrich nursing education. At the same time, the findings should be interpreted cautiously because of the small sample, questionnaire attrition, and the retrospective reconstruction of matched pre-post cases from archived exports. These issues do not negate the value of the program, but they do limit the strength of causal inference and the precision of statistical estimates. Even with these caveats, the program offers a practical model for internationalisation at home and for embedding culturally responsive, globally oriented learning within nursing curricula. As healthcare systems become increasingly multicultural and interconnected, COIL represents a feasible strategy for preparing future nurses and midwives for cross-cultural practice while reducing the barriers associated with physical mobility.

## Supplemental Material

sj-pdf-1-wjn-10.1177_01939459261488136 – Supplemental material for Developing Global Competence From Participation in an Online Collaborative Multicultural Learning Experience Supplemental material, sj-pdf-1-wjn-10.1177_01939459261488136 for Developing Global Competence From Participation in an Online Collaborative Multicultural Learning Experience by Gulzar Malik, Arkers Kwan Ching Wong, Jacqueline Parkin, Jacqueline Johnston, Kusrini Kadar and Katherine Frodsham Webster in Western Journal of Nursing Research
