# Supplementary material for: Developing Global Competence From Participation in an Online Collaborative Multicultural Learning Experience
Source: West J Nurs Res. 2026 Oct 13;48(12):1279–88. doi: 10.1177/01939459261488136 (PMC13748480; doi:10.1177/01939459261488136)
Supplement: Supplementary file 1 [file sj-pdf-1-wjn-10.1177_01939459261488136.pdf]

Case Study

Arthur, Josie and their baby Alicia

Josie became pregnant unexpectedly having previously been told that because of an underlying health condition she would never be able to conceive naturally. The pregnancy was initially a shock to both Josie and Arthur, but they were also overjoyed and began to prepare for the arrival of their daughter. Josie had a relatively uneventful pregnancy and continued to work in her busy role as a support worker in an older peoples nursing home. Arthur was concerned about Josie's heavy workload, but Josie insisted on continuing to work through the latter stages of her pregnancy.

When Josie went into labour, initially, things were going smoothly. However, a pre-existing allergy to an anaesthetic agent and complications during labour led to a fraught conversation between healthcare professionals and Josie where she was told that there was a chance she might not survive the birth. Arthurs words are as follows:

I'm sat in a little chair in the corner of the hospital thinking, so she's' basically just told me that my partner could die, that's basically the words: you might not make it through and there wasn't any, "are you ok with this?" "Do you want any input into this?" I was just like on the back burner a bit, the whole hospital experience wasn't very nice,

Alicia was born but was admitted to neonatal intensive care for two weeks due to lung problems and difficulty with feeding. On discharge home, the feeding problems continued, and Arthur and Josie had to pay for special milk due to Alicia's digestive problems.

There were very long nights and days with a baby who was very unhappy and seemingly in discomfort. Arthur worked long days and was only entitled to two weeks of paternity leave. His employers were very sympathetic and gave him extra leave whilst Alicia was an inpatient in the NICU.

More of Arthur's words:

So, I did get the two weeks at home but that was two weeks of crying, she couldn't poo, so we were up at 2am screaming the house down because she couldn't do a poo and it was the most stressful two weeks of my life and I thought, that's it now, I've got to go back to work and leave it all to you. You've got to do this on your own now, no sleep. I start work at four in the morning so I can be up all night helping you.

Consider the risks and protective factors for this family in relation to their mental health

1. What do you think that Arthur and Josie are at risk of?
2. What are the societal factors/cultural considerations which impact upon this family's mental health?
3. In your country, how Arthur would be supported and what services would be available to the family?
4. How is this clinical scenario typically managed in your country, and what cultural factors influence that practice?
5. What similarities and differences do you observe across your group members' contexts?
